# Individualized ventilatory management in patients with COVID-19-associated acute respiratory distress syndrome

**DOI:** 10.1016/j.rmcr.2021.101433

**Published:** 2021-05-31

**Authors:** Hiroki Taenaka, Takeshi Yoshida, Haruka Hashimoto, Hirofumi Iwata, Yukiko Koyama, Akinori Uchiyama, Yuji Fujino

**Affiliations:** The Department of Anesthesiology and Intensive Care Medicine, Osaka University Graduate School of Medicine, Suita, Japan

**Keywords:** COVID-19, ARDS, Mechanical ventilation, PEEP, EIT

## Abstract

Due to the coronavirus disease 2019 pandemic, the number of coronavirus disease 2019-associated acute respiratory distress syndrome is rapidly increasing. The heterogeneity of coronavirus disease 2019-associated acute respiratory distress syndrome contributes to the complexity of managing patients. Here we described two patients with coronavirus disease 2019-associated acute respiratory distress syndrome showing that the bedside physiological approach including careful evaluation of respiratory system mechanics and visualization of ventilation with electrical impedance tomography was useful to individualize ventilatory management.

## Abbreviations

COVID-19: Coronavirus disease 2019
ARDS: Acute respiratory distress syndrome
PEEP: Positive end expiratory pressure
EIT: Electrical impedance tomography
ROI: Region of interest

## 1 Introduction

Due to the coronavirus disease 2019 (COVID-19) pandemic, 70% of critically ill patients with COVID-19 develop acute respiratory distress syndrome (ARDS) [1]. Most of patients with ARDS due to COVID-19 require invasive mechanical ventilation [2]. Of note, previous papers pointed out COVID-19-associated ARDS had distinctive physiological characteristics that were apart from common ARDS, in terms of the dissociation between the severity of hypoxemia and lung recruitablity (*i.e.*, response to positive end expiratory pressure (PEEP) or position) [[3], [4], [5]]. Therefore, personalized ventilatory strategy may be required in COVID-19-associated ARDS according to respiratory system mechanics, with a view of minimizing ventilator-induced lung injury.

Here we describe two cases of patients with ARDS due to COVID-19 who had different response to PEEP and position. The careful evaluation of respiratory system mechanics in combination of different PEEP and position was useful to optimize ventilatory strategy by using electrical impedance tomography (EIT).

## 2 Case presentation

The current study was approved by the Ethics committee for Clinical Studies, Osaka University Hospital, Suita, Japan (No.20039). Patients were assigned to each of four conditions in a sequential order:

• High PEEP, Supine;
• Low PEEP, Supine;
• High PEEP, Prone;
• Low PEEP, prone.

**Fig. 1:**
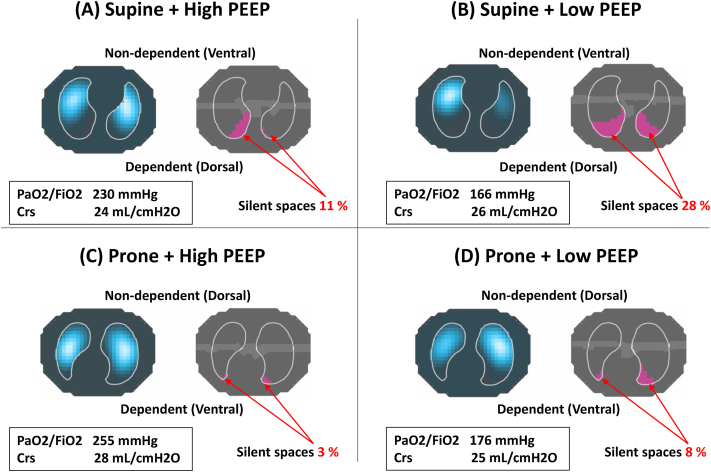
Respiratory variables, distribution of ventilation, and ‘silent spaces’ in ***Case 1*** (A) High PEEP, Supine; (B) Low PEEP, Supine; (C) High PEEP, Prone; and (D) Low PEEP, Prone. In low PEEP + supine position, oxygenation was worst and the largest amount of ‘ silent spaces’ was observed, suggesting a massive lung collapse in dependent lung regions (B). High PEEP improved oxygenation, reduced the amount of ‘ silent spaces’ (A). Prone position by itself reduced the amount of silent spaces in dependent lung regions without increasing PEEP (D). The combination of high PEEP with prone position achieved highest oxygenation, least amount of ‘ silent spaces’ (C). PEEP = positive end-expiratory pressure; Crs = respiratory system compliance.

All patients were ventilated with assisted volume-controlled mode, targeting V_T_ of 6 ml/kg predicted body weight. EIT (SenTec AG, Landquart, Switzerland) monitoring was initiated to evaluate the distribution of ventilation. ‘Silent spaces’ was defined as the region of interest (ROI) showing impedance changes were less than 10% of maximal impedance changes during tidal ventilation [6]. The amount of silent spaces were expressed as a percentage of the entire ROI [6] (Fig. 1). All respiratory parameters were measured in each position.

### 2.1 Case 1

A 61-year-old female was transferred to our ICU and intubated due to hypoxemia twelve days after laboratory-confirmed COVID-19 infection. The criteria of moderate ARDS (PaO2/FiO2 ratio: 166 mmHg) was met according to Berlin definition at PEEP of 5cmH_2_O [7].

In supine position, oxygenation was worse at low PEEP and silent spaces reached at 28% in dependent lung regions, suggesting a massive alveolar collapse and high PEEP improved oxygenation and reduced the amount of silent spaces to 11%. Prone position reduced the amount of silent spaces in the dependent lung regions at low PEEP (*vs*. low PEEP, supine). The combination of high PEEP with prone position achieved highest oxygenation, highest respiratory system compliance, least amount of silent spaces (Fig. 1). Therefore, we applied PEEP of 15cmH_2_O and prone position was continued for three days. At Day 7, she was successfully extubated and discharged from ICU at Day 13.

### 2.2 Case 2

A 73-year-old male was transferred to our ICU and intubated due to hypoxemia eight days after laboratory-confirmed COVID-19 infection. The criteria of mild ARDS (PaO2/FiO2 ratio: 243 mmHg) was met according to Berlin definition at PEEP of 5cmH_2_O [7].

**Fig. 2:**
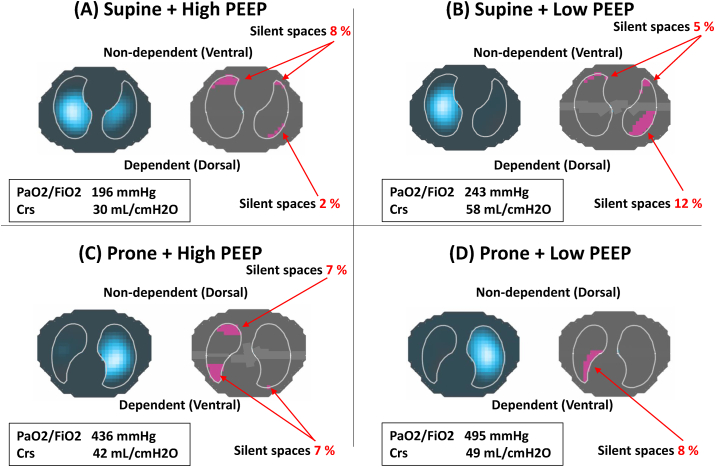
Respiratory variables, distribution of ventilation, and ‘silent spaces’ in ***Case 2*** (A) High PEEP, Supine; (B) Low PEEP, Supine; (C) High PEEP, Prone; and (D) Low PEEP, Prone. Regardless of position, the amount of ‘ silent spaces’ in non-dependent lung increased, oxygenation deteriorated, and respiratory system compliance reduced by increasing PEEP, suggesting PEEP induced overdistension, (A, C). Prone position at low PEEP had the least amount of ‘silent spaces’ in non-dependent lung, achieving the highest value of oxygenation (D). PEEP = positive end-expiratory pressure; Crs = respiratory system compliance.

In contrast to ***case 1***, in both positions, the amount of silent spaces in non-dependent lung increased, oxygenation deteriorated, and respiratory system compliance reduced by increasing PEEP, all of which suggest higher PEEP overdistended non-dependent lung regardless of position. Prone position at low PEEP (*vs.* supine at low PEEP) reduced the amount of silent spaces in the dependent lung from 12% to 8% and it also had the least amount of silent spaces in the non-dependent lung, achieving the highest value of oxygenation (Fig. 2). Therefore, we applied low PEEP of 7cmH_2_O and prone position was continued for three days. At Day 4, she was successfully extubated and discharged from ICU at Day 6.

## 3 Discussion

Here we described two patients with COVID-19-associated ARDS showing that the tailored physiological approach including careful evaluation of respiratory system mechanics and visualization of ventilation with EIT was useful to optimize ventilatory management.

The heterogeneity of COVID-19-associated ARDS contributes to the complexity of managing patients in ICU [4]. What level of PEEP to use (*i.e.,* high or low) and what position to use (*i.e.,* supine or prone) are difficult to choose without bedside physiology [8,9]. Here we performed a simple bedside technique to tailor ventilatory management in patients with COVID-19-associated ARDS. *First*, in both cases, we found that prone position itself was effective to improve oxygenation and to reduce the amount of alveolar collapse (suggested by smaller amount of ‘silent spaces of dependent lung’) without increasing PEEP even though PaO2/FiO2 ratio was over 150 mmHg at PEEP of 5cmH_2_O in supine position. These are unpredicted findings since prone position is not recommended in patients whose oxygenation are more than 150 mmHg [10]. *Second*, response to PEEP was found to be different in each patient. The visualization of ‘silent spaces of non-dependent lung’ with EIT was helpful to suspect if increasing PEEP induced hyperinflation.

The current case report suggests that the application of such a tailored physiological approach may help physicians to identify PEEP level and position adequate to each patient with COVID-19-associated ARDS.

## 4 Conclusions

The bedside physiological approach including careful evaluation of respiratory system mechanics and visualization of ventilation with EIT was useful to individualize ventilatory management, *i.e.*, PEEP and position, in patient with COVID-19-associated ARDS.

## Declarations of interest

The authors have no financial or non-financial competing interests related to this case report.

## Funding

There is no funding associated with this case report.

## Declaration of competing interest

The authors declare that they have no known competing financial interests or personal relationships that could have appeared to influence the work reported in this paper.

## References

[1] Yang X., Yu Y., Xu J., Shu H., Xia J., Liu H. (2020). Clinical course and outcomes of critically ill patients with SARS-CoV-2 pneumonia in Wuhan, China: a single-centered, retrospective, observational study. Lancet Respir. Med..

[2] Cummings M.J., Baldwin M.R., Abrams D., Jacobson S.D., Meyer B.J., Balough E.M. (2020). Epidemiology, clinical course, and outcomes of critically ill adults with COVID-19 in New York City: a prospective cohort study. Lancet.

[3] Pan C., Chen L., Lu C., Zhang W., Xia J.A., Sklar M.C. (2020). Lung recruitability in SARS-CoV-2 associated acute respiratory distress syndrome: a single-center, observational study. Am. J. Respir. Crit. Care Med..

[4] Gattinoni L., Chiumello D., Caironi P., Busana M., Romitti F., Brazzi L. (2020). COVID-19 pneumonia: different respiratory treatments for different phenotypes?. Intensive Care Med..

[5] Gattinoni L., Coppola S., Cressoni M., Busana M., Rossi S., Chiumello D. (2020). Covid-19 does not lead to a "Typical" acute respiratory distress syndrome. Am. J. Respir. Crit. Care Med..

[6] Ukere A., Marz A., Wodack K.H., Trepte C.J., Haese A., Waldmann A.D. (2016). Perioperative assessment of regional ventilation during changing body positions and ventilation conditions by electrical impedance tomography. Br. J. Anaesth..

[7] Ranieri V.M., Rubenfeld G.D., Thompson B.T., Ferguson N.D., Caldwell E., Fan E. (2012). Acute respiratory distress syndrome: the Berlin Definition. JAMA.

[8] Goligher E.C., Kavanagh B.P., Rubenfeld G.D., Ferguson N.D. (2015). Physiologic responsiveness should guide entry into randomized controlled Trials. Am. J. Respir. Crit. Care Med..

[9] Papazian L., Paladini M.H., Bregeon F., Thirion X., Durieux O., Gainnier M. (2002). Can the tomographic aspect characteristics of patients presenting with acute respiratory distress syndrome predict improvement in oxygenation-related response to the prone position?. Anesthesiology.

[10] Guerin C., Reignier J., Richard J.C., Beuret P., Gacouin A., Boulain T. (2013). Prone positioning in severe acute respiratory distress syndrome. N. Engl. J. Med..

